# Seasonal patterns of mercury bioaccumulation in lobsters (*Homarus americanus*) from Maine

**DOI:** 10.20935/acadbiol7544

**Published:** 2025-02-26

**Authors:** Dan Stoicov, Carolina A. Bonin, Andre J. van Wijnen, Eric A. Lewallen

**Affiliations:** 1 Department of Biological Sciences, Hampton University, Hampton, VA 23668, USA.; 2 Department of Marine and Environmental Sciences, Hampton University, Hampton, VA 23668, USA.; 3 Department of Biochemistry, University of Vermont, Burlington, VT 05405, USA.

**Keywords:** biomagnification, coastal marine ecology, ecotoxicology, fishery biology, methylmercury

## Abstract

Mercury (Hg) pollutes marine ecosystems and accumulates in benthic species. This ecological case study investigated the temporal accumulation of Hg in American lobster (*Homarus americanus; H. Milne Edwards, 1837*) from coastal Maine (Casco Bay, ME, USA). We analyzed total Hg levels in legal-sized lobsters (carapace length: 8.255–12.5 cm; n = 34) collected during the early (May–July 1) or late (July 15–October) recreational harvest seasons. Morphometric data show that body size correlates with body weight (R^2^ = 0.76; p < 0.001), and average body sizes were similar in early and late seasons. The average chelipod size was ~7% larger in male lobsters (p < 0.02), reflecting sexual dimorphism. Hg levels in select tissues from boiled lobsters were analyzed using atomic absorption spectroscopy. Hg in ambient water was undetectable, indicating that Hg in tissues reflects bioaccumulation. Hg content correlated with the lengths (cm) and weights (g) of cephalothorax, carapace, chelipod, and hepatopancreas in both male and female lobsters. Total Hg levels in most tissues were within safe and acceptable limits for human consumption (<0.2 ppm). Compared to late-season lobsters, early-season lobsters had significantly higher Hg levels in tail (~55% increase; 0.130 ppm vs. 0.084 ppm; p < 0.05) and hepatopancreas tissues (~29% increase; 0.099 ppm vs. 0.077 ppm; p < 0.05), suggesting that seasonal factors influence Hg content (e.g., spring river runoff, lobster migration, inert biological cycles). Observed seasonal fluctuations in lobster Hg levels may inform future strategies for mitigating pollution in coastal marine ecosystems.

## 1. Introduction

Mercury (Hg) is a naturally occurring element found in the lithosphere that exists in elemental or organic forms, with varying degrees of solubility in marine ecosystems. Environmental Hg is a principal public health concern and originates mostly from human activities (e.g., coal mining, fossil fuel combustion). The most ubiquitous mercurial compound that poses a threat to public health is methylmercury (MeHg). MeHg is formed by anaerobic bacteria that transform inorganic Hg into an organometallic cation [CH_3_Hg]^+^ in aquatic environments. For almost 50 years, Hg content in lobsters harvested for human consumption has been recognized as a potential health concern [1]. Human exposure to Hg occurs primarily through MeHg-contaminated seafood [2], dental amalgams, and occupational exposure. Chronic exposure to Hg can lead to neurological, digestive, renal, endocrine, muscular, and immunological dysfunctions [3]. MeHg is considered a neurotoxin that is potentially harmful to fetal brain development [4]. Exposure to Hg in pregnant women can lead to pregnancy complications and developmental disorders in infants. Hg also affects lobsters themselves, particularly in tissues (e.g., gills) required for osmoregulation [5, 6]. The Maine Center for Disease Control and Prevention considers <200 ng of Hg per gram of meat (0.2 parts per million [ppm]) to be safe. The global increase in Hg pollution renders coastal ecosystems and fisheries vulnerable to the potential impact of increased bioorganic MeHg exposure in seafood products.

The increasing bioavailability of MeHg results in its bioaccumulation in individual metazoan species and subsequent biomagnification in ecological trophic systems, with humans being particularly vulnerable as apex predators [7–9]. Seafood represents a primary source of Hg exposure in humans in the form of MeHg, as observed in multiple marine habitats [10–12]. In marine environments, MeHg concentrations vary by species, with species at higher trophic levels (e.g., apex predators, omnivores, and detritivores) typically accumulating the highest MeHg content. Long-lived marine arthropods such as the American lobster (*H. americanus*), which can live for over 100 years, serve as excellent indicator species for MeHg bioaccumulation, whereas short-lived species such as shrimp (lifespan <5 years) may be less informative [13, 14]. Lobsters ingest toxic contaminants as omnivores and scavengers of dead organic material (detritus) in benthic marine habitats. However, *H. americanus* is considered one of the safest species for consumption in relation to Hg content (0.107 ppm) [15].

The American lobster industry is one of the oldest continually operating industries in North America, dating back to the 1600s. In 2022, the industry harvested 120 million pounds of lobster, with a total ex-vessel value of $519 million [16]. Lobstering is a vital component of the U.S. economy in the Northeastern states and creates approximately 18,000 jobs annually in Maine (2021). Consumers are advised to refrain from eating tomalley (hepatopancreas), the soft green substance found in the body cavity of lobsters, as it often contains unsafe levels of Hg and other contaminants [17]. One example of Hg-related ocean pollution occurred in Maine in 2014, when the Department of Marine Resources (DMR) found that lobsters at the mouth of the Penobscot River contained high levels of Hg (0.293 ppm), leading to the closure of the area for lobstering. The Maine DMR conducts testing for toxic chemicals (including Hg) in the marine environment every five years under the Surface Water Ambient Toxics (SWAT) monitoring program but does not regularly evaluate Hg levels in lobster tissues throughout the entire fishing season.

Analysis at a single five-year interval provides only a snapshot of Hg concentrations in lobsters. However, a broad range of biological and environmental factors may collectively result in more rapid temporal fluctuations in Hg levels in marine animal tissues and coastal waters. The bioaccumulation of Hg in lobster tissues may be influenced by multiple biological parameters, including growth (molting), migration, and reproductive cycles, as well as interactions with other species throughout the trophic system. Environmental factors such as spring ice melt, rain runoff, agricultural runoff, and increased economic and recreational activities during warmer months may also alter Hg levels. Therefore, we hypothesized that Hg levels in lobster tissues may exhibit seasonal variation. To test this hypothesis, we conducted a chronological analysis of total Hg levels in lobsters throughout the fishing season. We assessed whether Hg levels in lobsters were related to morphometric parameters (e.g., claw length and body shell size) and anatomical location in both the early and late stages of the lobster harvest season.

## 2. Materials and methods

### 2.1. Collection of lobster specimens

Lobsters were collected in the Casco Bay waters (43.68°N, 70.18°W) of the Gulf of Maine during the regular lobster harvest season from May 31 to October 1, 2023, under a Maine recreational license (ME 13000, issued to Dan Stoicov). Lobsters were caught using standard plastic-coated wire pots with a wire spacing designed to reduce the probability of marine by-catch. The pots feature a compartmental design with two funneled heads that permit entry while preventing the escape of lobsters. Lobsters were lured into the pots using bait composed of a mixture of chum derived from locally caught fish, including inshore Atlantic Pollock (*Pollachius virens*), Atlantic Menhaden (*Brevoortia tyrannus*), and Atlantic Mackerel (*Scomber scombrus*). Upon retrieval of the pots, we adhered to local catch-and-release regulations by rapidly screening body size based on carapace (shell) length (i.e., the distance from the eye socket to the rear edge of the cephalothorax). Lobsters with shell sizes outside the legal collection range (8.255–12.5 cm) were immediately released along with any by-catch. Fertile female lobsters carrying eggs were tail-notched and immediately released regardless of size, along with previously tail-notched females without eggs, in compliance with state regulations. Legal-sized lobsters were collected every 2–4 weeks (4–6 per time point) for 34 lobsters (male: n = 22; female: n = 12). The requirement to release fertile female lobsters largely accounts for the resulting sex bias in the sample.

### 2.2. Morphometric analysis of lobster specimens

Each lobster was labeled and weighed (in grams). The lengths of the cephalothorax carapace, chelipod (crushing claw), and tail were measured in centimeters. The sex, reproductive status, and shell type (soft or hard) were recorded for each individual. All lobsters were sacrificed in boiling water for 15 min, allowed to cool, and dissected for the acquisition of biopsies for chemical analysis. Anatomically distinct specimens (~10 g each) were collected as wet tissues from the crushing claws, tails, and tomalley. Specimens were placed in 10 ml Kimberly Fisher test tubes and stored at −20°C prior to testing for total Hg content.

### 2.3. Hg analysis in lobster specimens

Lobster tissue specimens were tested for total Hg content by atomic absorption spectroscopy using a Leco AMA254 Advanced Mercury Analyzer (St. Joseph, MI) at Exact Scientific Services laboratories (Ferndale, WA). This equipment combusts samples in the presence of oxygen to release Hg vapor and eliminates interfering substances (e.g., halogens and ash) using a catalytic device. Hg is collected by adsorption onto a gold amalgamator trap. The captured Hg vapor is then released by rapid heating and analyzed via absorption spectrophotometry. This instrument has a detection range of 0.05–500 ng, a precision of 0.25 ppb, and a readability of 0.1 ppb, as well as standard sample calibration and certification functions that comply with the U.S. Environmental Protection Agency. Hg levels are reported in ppm of wet weight for each tissue type.

### 2.4. Statistical analysis

Quantitative morphometric and chemical data were examined for statistical significance using Student’s t-tests, linear regression analysis, and calculation of Pearson’s coefficients in Microsoft Excel (Redmond, WA) [18]. The null hypothesis (no difference in parameters) was rejected at a significance level of p < 0.05.

## 3. Results

### 3.1. Geographic origin of the lobster population

**Figure 1 •:**
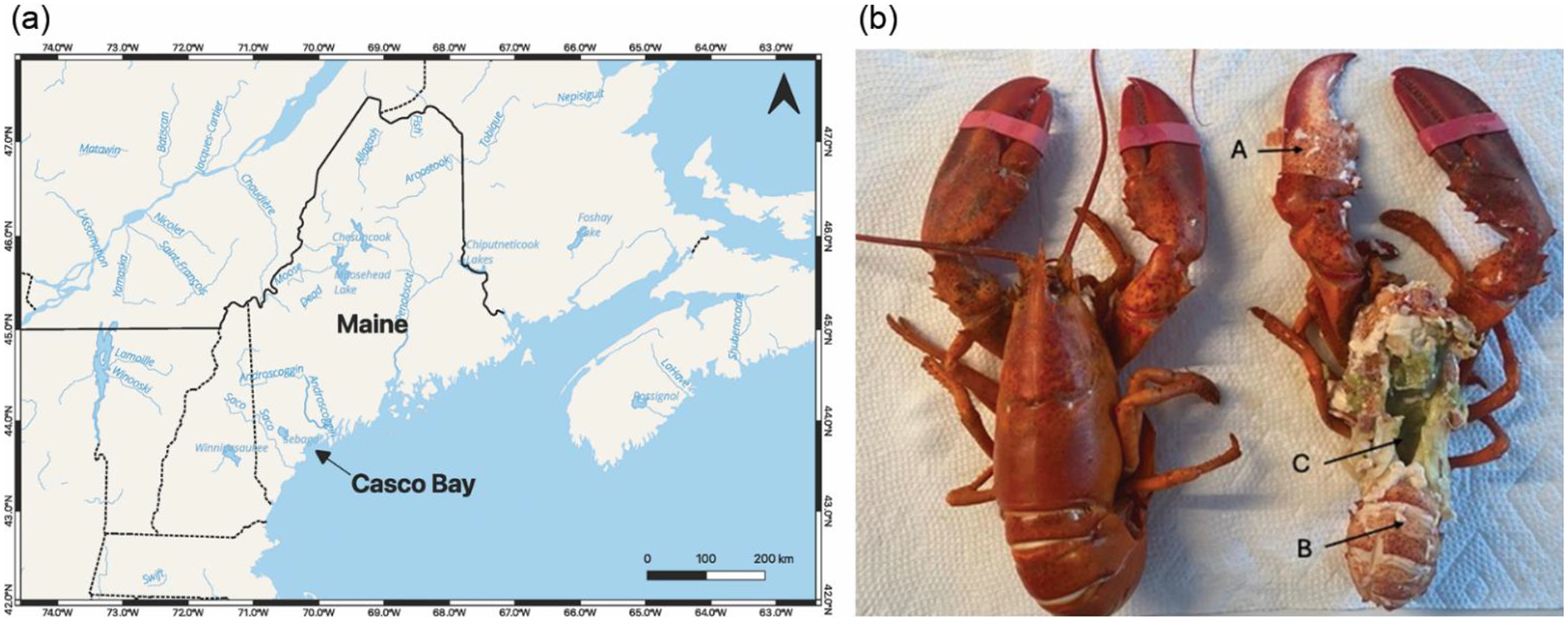
(a) A map of the location (Casco Bay, ME) where American lobsters were collected for this study. This map was generated using freely available QGIS software for Mac OS (v3.40) [20]. (b) A photo indicating American lobster tissues collected for Hg analysis. (A) Crusher claw. (B) Tail. (C) Tomalley (hepatopancreas).

Lobsters used in this study were harvested in Casco Bay, Gulf of Maine, throughout the 2023 fishing season (May 2023–October 2023). Casco Bay lies between the Two Lights lighthouses in Cape Elizabeth, ME, Cape Small in Phippsburg, ME, and north of Halfway Rock Island (Figure 1). The bay encompasses 14 distinct coastal communities, including Portland, the largest city in Maine. The waters of Casco Bay serve as a navigation route for oil tankers, cruise ships, fishing vessels, container ships, ferries, water taxis, and recreational vessels. Multiple rivers, including the Fore, Presumpscot, Harraseeket, Royal, and Cousins, empty directly into Casco Bay. Casco Bay is an estuary and a diverse habitat that supports approximately 850 marine species and 150 species of birds [19].

### 3.2. Morphometric data of the lobster cohort

**Figure 2 •:**
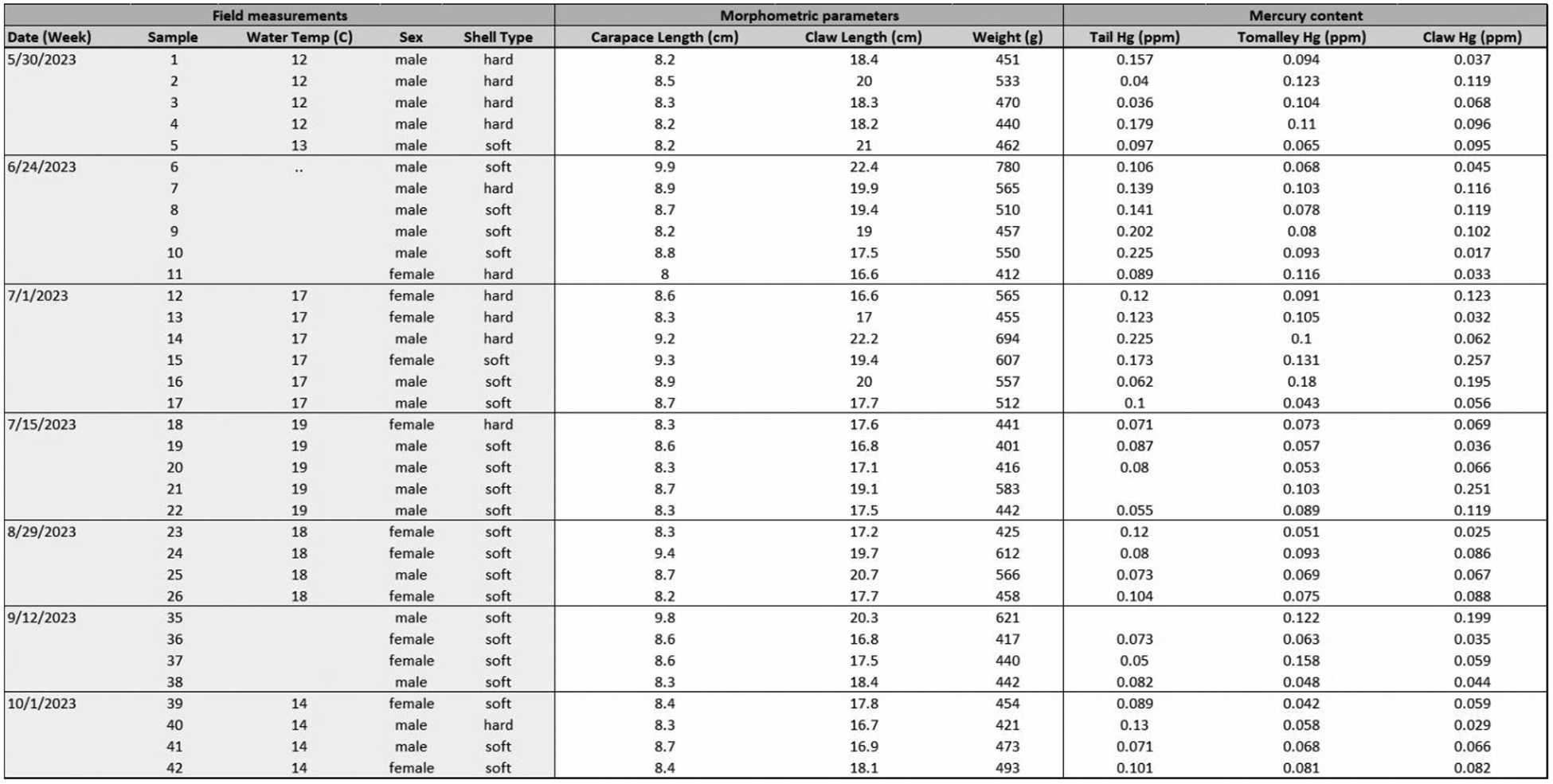
Sampling data for American lobsters collected in Casco Bay, ME. Field measurements including dates, sample identification numbers, local seawater temperatures, sex, shell type, morphometric data (carapace length, claw length, and body weight), and Hg measurements for each tissue type (tail, tomalley, and claw) are shown. Any female lobster with visible eggs was released in the field and excluded from the study.

A total of 34 lobsters were harvested during the 2023 fishing season on the following dates: 05/30 (5 lobsters), 06/24 (6 lobsters), 07/01 (6 lobsters), 07/15 (5 lobsters), 08/29 (4 lobsters), 09/12 (4 lobsters), and 10/01 (4 lobsters). The fishing season was divided into the early season (May–July 1) and the late season (July 15–October). For each lobster, we recorded a range of physical parameters including sex (male/female), reproductive status (no eggs visible because gravid females were released), shell type (soft vs. hard), shell length (cm), claw length (cm), and total weight (g). Our sampled lobster cohort included only specimens that were bigger than 8 cm and smaller than 12.5 cm to comply with local catch-and-release criteria (Figure 2).

**Figure 3 •:**
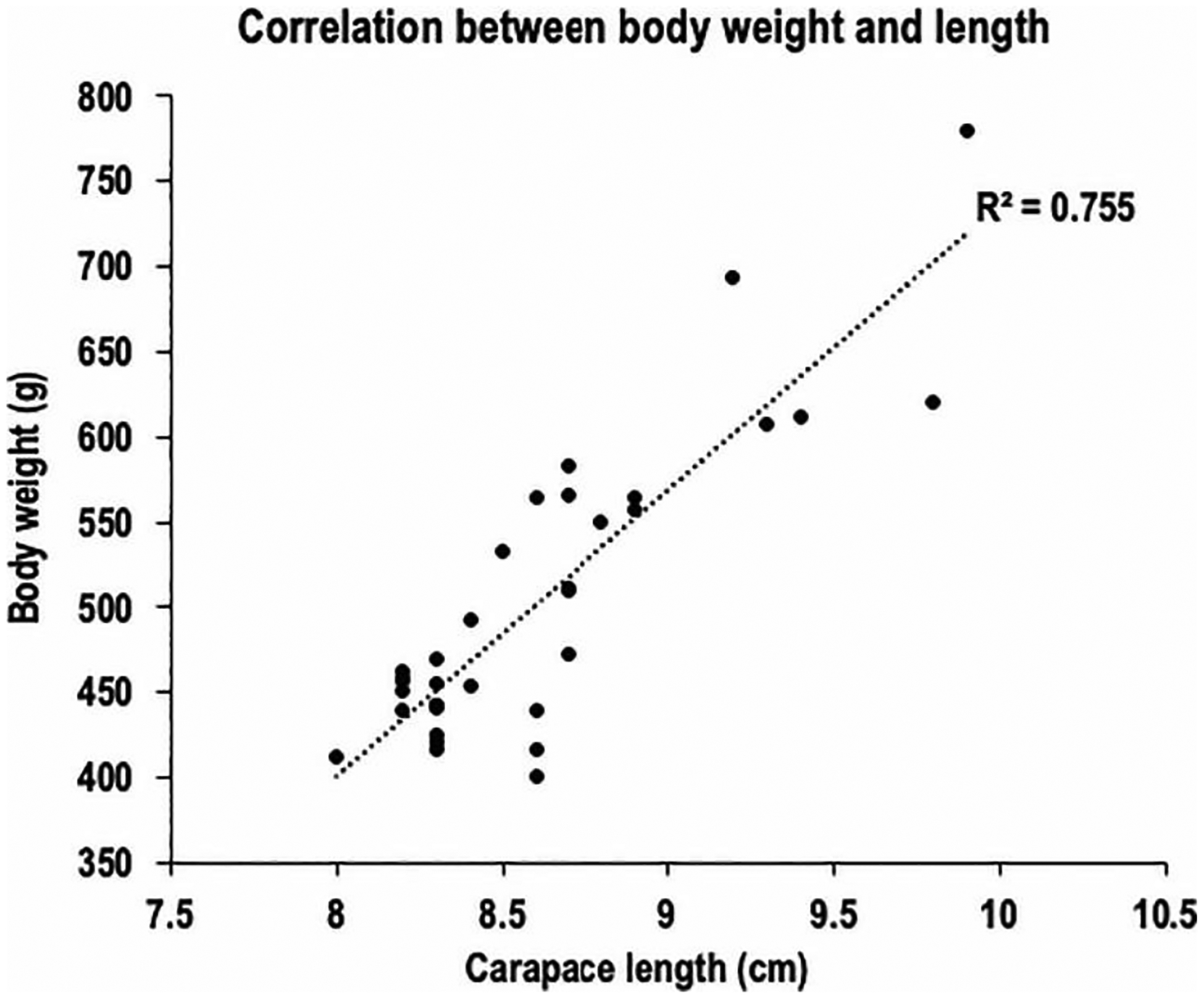
Significant positive correlation between carapace length and body weight of American lobsters collected in Casco Bay, ME (n = 34; Pearson’s r = 0.87; R^2^ = 0.76).

**Figure 4 •:**
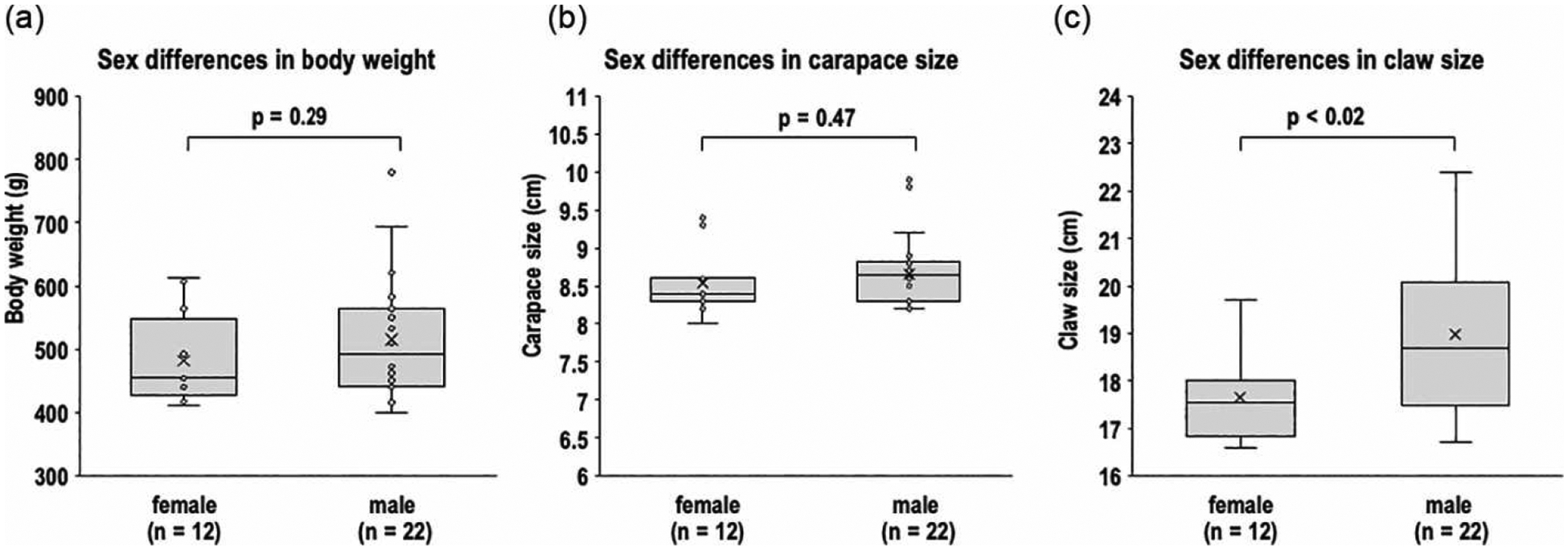
Comparisons of body weight (a), carapace size (b), and claw size (c) in female (n = 12) versus male American lobsters (n = 22). Middle horizontal lines in the gray boxes represent medians, “x” represents means, whiskers represent ± standard error, and the small circles outside of the boxes represent outliers. Note that the only significant morphometric differences between sexes were observed in claw size.

To define our lobster cohort, we analyzed weight, shell length, and claw length for each sex in both the early and late seasons. As expected, the morphometric data showed a positive linear correlation between lobster body size (carapace length) and overall body weight (R^2^ = 0.76; Pearson’s r = 0.87) (Figure 3). We also assessed whether morphometric parameters exhibited sex-related differences. No significant sex-related differences (p > 0.05) were observed for carapace size or body weight (Figure 4a and b). However, comparison of claw sizes revealed that male lobsters, on average, had claws that were 7.4% larger than those of females (p < 0.02) (Figure 4c). This male-specific increase in claw size is anatomically distinct. The modest sexual dimorphism in claw size is consistent with previous observations in Norwegian lobster populations and may be linked to claw use in courting displays [21]. Because we did not observe overt sex-biased differences in external anatomy (e.g., >10% difference in length or size), we combined morphometric data for male and female lobsters to increase cohort size.

**Figure 5 •:**
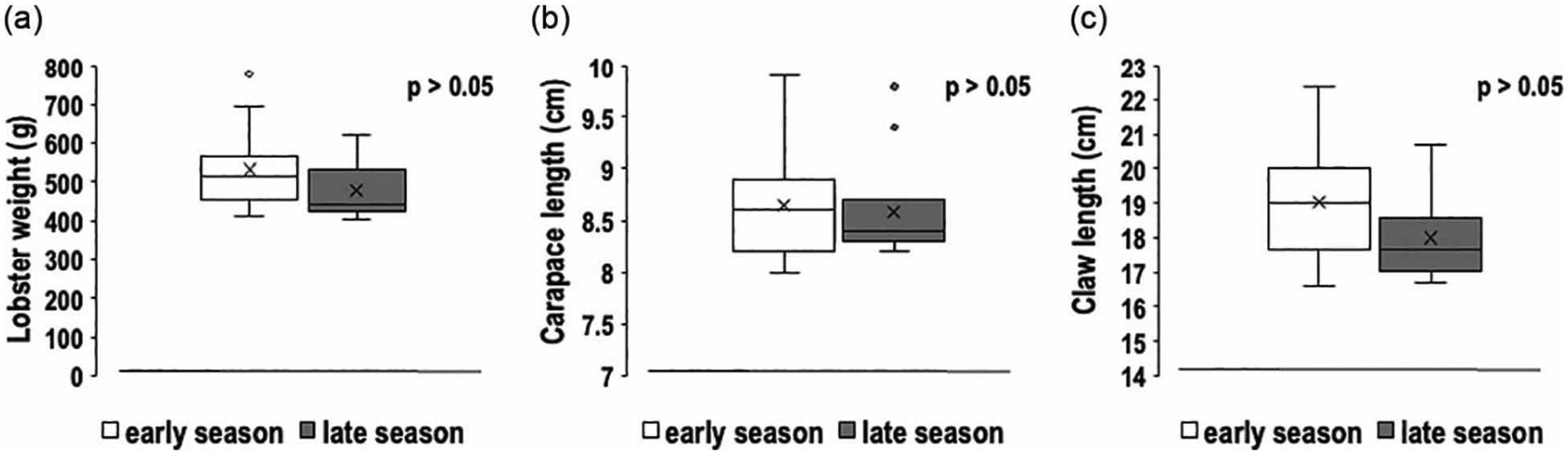
Comparisons of mean weight (a), carapace length (b), and claw length (c) of American lobsters collected in early and late seasons. Middle horizontal lines in the gray boxes represent medians, “x” represents means, whiskers represent ± standard error, and the small circles outside of the boxes represent outliers. Note that none of the differences were significant.

We analyzed our morphometric data to assess potential seasonal differences in lobster size. The data show that lobster weight exhibited a modest numerical difference between the early and late seasons (531 g vs. 477 g), but this difference was not statistically significant (p > 0.05) (Figure 5a). Similarly, the mean carapace length was 8.64 cm in the early season and 8.58 cm in the late season, but this difference was also not statistically significant (p > 0.05) (Figure 5b). Claw lengths did not differ significantly, with average claw sizes of 19 cm in the early season and 18 cm in the late season (p > 0.05) (Figure 5c). The similarities in these measurements over time indicate that the anatomical sizes and body weights of lobsters in the early and late seasons are comparable.

### 3.3. Analysis of Hg levels in lobster tissues

**Figure 6 •:**
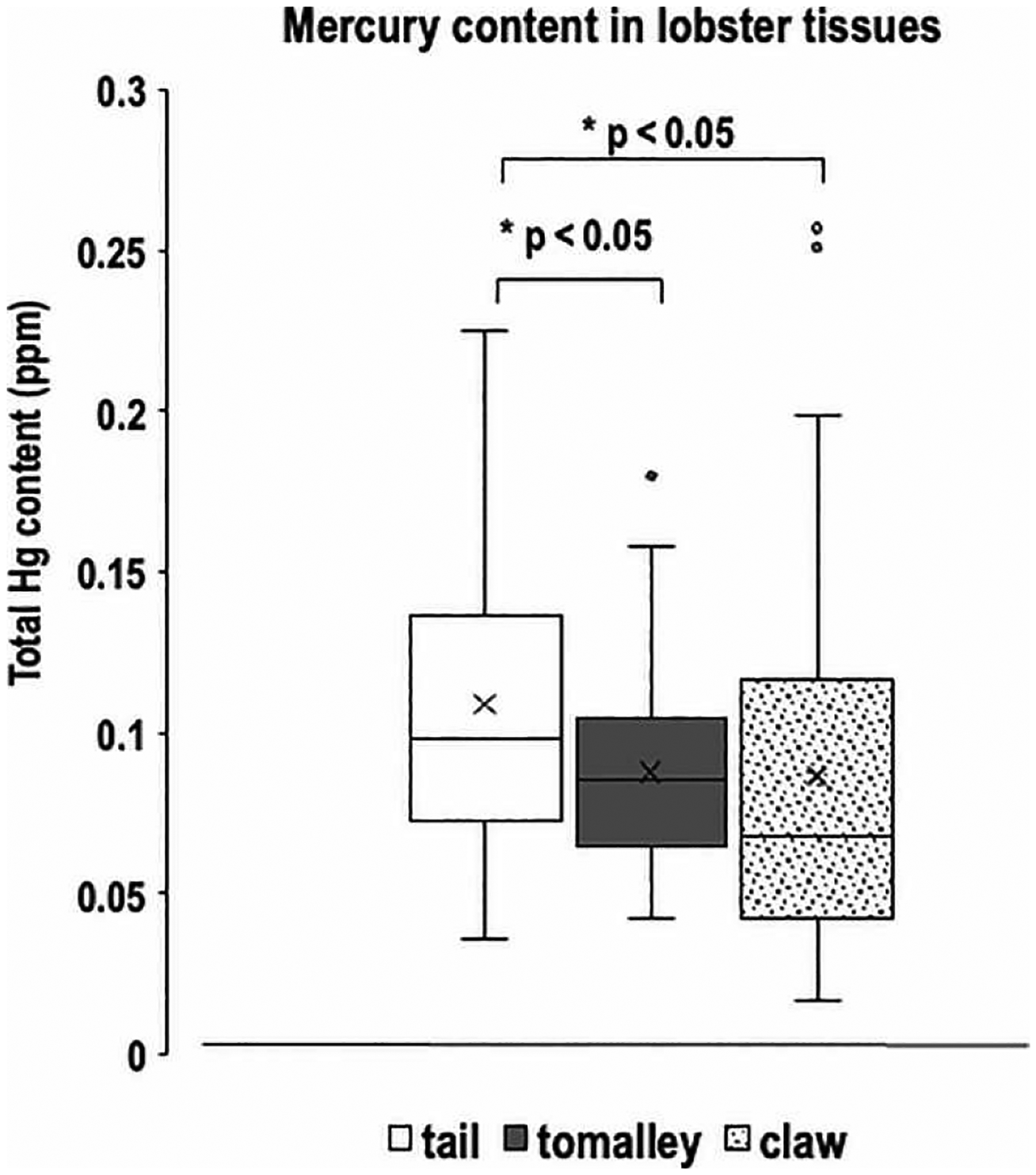
Mean total Hg in tail, tomalley, and claw tissues of American lobsters. Middle horizontal lines in the gray boxes represent medians, “x” represents means, whiskers represent ± standard error, and the small circles outside of the boxes represent outliers.

Previous studies have identified multiple routes through which exogenous Hg can enter lobster tissues [1]. The majority of ingested Hg is initially processed in the tomalley, where it has a half-life of three weeks before accumulating in muscle tissue (e.g., the tail). Although Hg in ambient water can be absorbed by tail tissue [1], ambient Hg levels are negligible compared with ingested Hg. To determine whether Hg accumulates at detectable levels in lobster tissues from the Gulf of Maine, we analyzed samples using atomic absorption spectroscopy. This method combusts the samples, collects the resulting Hg vapor, and quantifies it. We assessed the mean total Hg levels in different body parts of all sampled lobsters, including the tail, tomalley, and claw (Figures 1b and 6). For comparison, total Hg levels in surface water samples collected throughout the year were below the level of detection (data not shown). Spectroscopic data revealed that lobster tails (always absent of eggs) contained significantly higher Hg levels (mean = 0.1088 ppm) than tomalley (mean = 0.088 ppm) (p < 0.05) and claw tissue (mean = 0.087 ppm) (p < 0.05) (Figure 6). There was no significant difference in Hg levels between tomalley and claw tissue. Thus, our results indicate that Hg concentration in lobster tail tissue was higher than in either tomalley or claw tissue. We also note that the mean Hg concentrations in all examined tissues remained well below the recommended safety limit of 0.2 ppm for human consumption.

**Figure 7 •:**
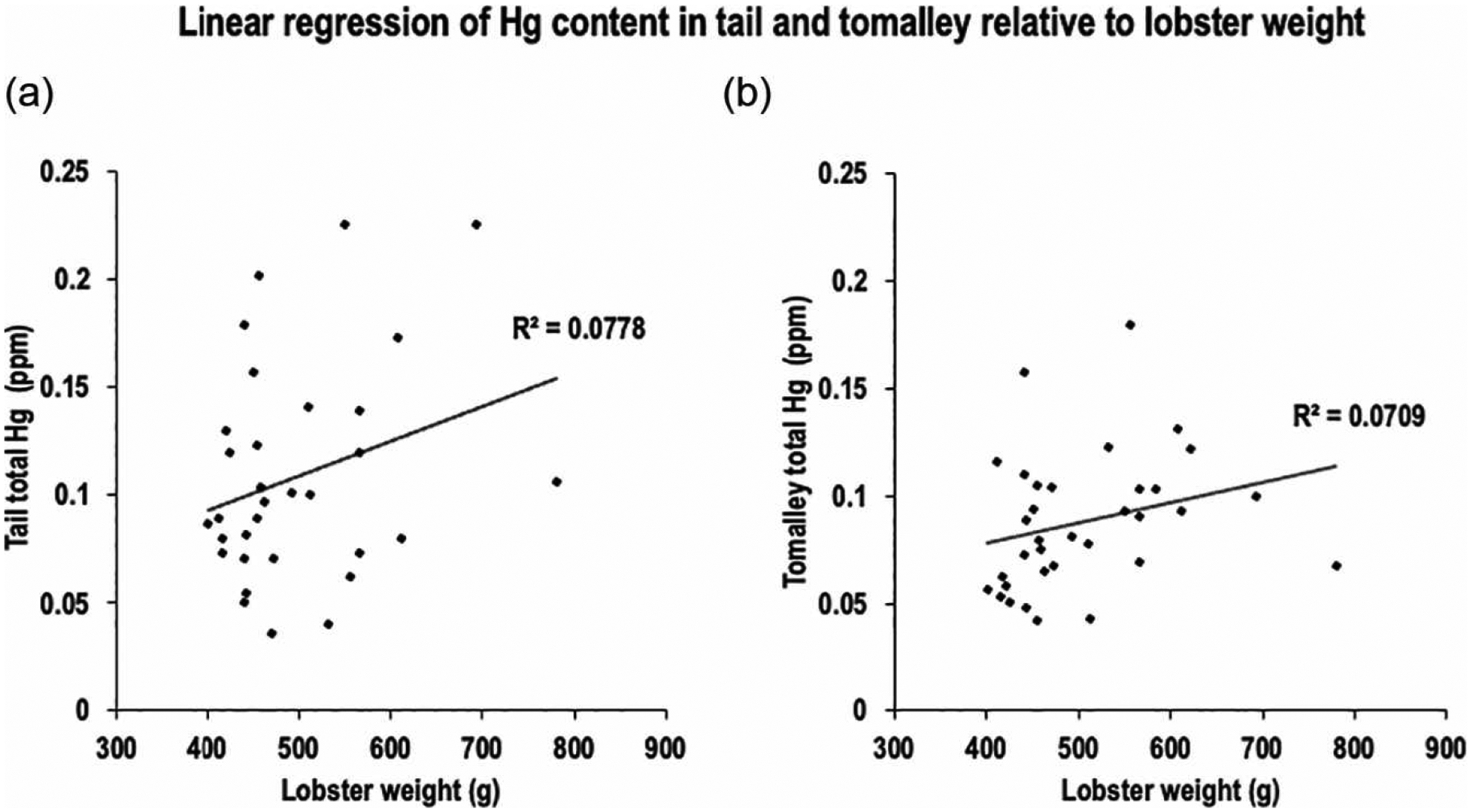
(a) The relationship between American lobster weight and tail total Hg content (Pearson’s r = 0.28; R^2^ = 0.0778; p > 0.05). (b) The relationship between American lobster weight and tomalley total Hg content (Pearson’s r = 0.267; R^2^ = 0.0709; p > 0.05).

Bioaccumulation of Hg may increase with age, and since lobster age correlates positively with total body weight, we tested whether Hg content of each tissue type increased with specimen weight. Linear regression analysis of the atomic absorption spectroscopy data showed no strong correlation between lobster weight and Hg levels in either tail tissue (Pearson’s r = 0.28, R^2^ = 0.078) or tomalley (Pearson’s r = 0.267, R^2^ = 0.071) (Figure 7). The best-fit line from linear regression for both tail and tomalley suggests a minor upward trend, indicating that larger lobsters (~800 g) may have about ~50% more Hg than smaller lobsters (~400 g). However, this pattern is inconsistent, as several smaller lobsters (<600 g) exhibited disproportionately high Hg levels (>0.15 ppm) (Figure 7). Although our data remain inconclusive, the absence of a clear correlation between age-related size and Hg levels indicates that bioaccumulation in lobsters depends on multiple stochastic factors.

Our finding that Hg concentrations are higher in tail muscle contradicts the expectation that Hg levels should be higher in tomalley. Tomalley, as a key metabolic organ, is expected to biologically concentrate Hg because it processes dietary components containing Hg from detritus and benthic prey. However, differences in Hg content between tail muscle and tomalley may be influenced by technical factors in sample processing that affect Hg detection efficiency. Previous studies have reported that Hg content can be lost from lobster tomalley during sample preparation [22]. Other researchers have recently proposed that solid-phase extraction methods may be preferable for extracting Hg from seafood products [23]. During this process, Hg is extracted from powdered seafood products by adding acetonitrile to prevent emulsions from forming and sulfuric acid to dissolve lipids. Therefore, this strategy would be of interest for long-term Hg-monitoring studies of powdered lobster tissues, including lipid-rich tomalley samples, that allow for archival and re-analysis.

In our case, the lower Hg levels in tomalley may result from increased Hg release into the surrounding water during boiling. Alternatively, the higher Hg concentration in tail muscle could be attributed to the high temperatures of boiling, which denature resilient Hg-binding proteins such as astaxanthin—the carotenoid responsible for the red coloration in crustaceans after cooking. Protein denaturation may increase Hg detection by increasing the availability of Hg for chemical analysis [22, 24]. Regardless of potential tissue-specific differences in Hg detection efficiency, this efficiency is expected to remain relatively constant within each tissue type across individual specimens. Considering additional contaminants (e.g., lithium [25]) that bioaccumulate in benthic species will also be relevant to the broader context of ecotoxicology and sample handling.

Our atomic absorption spectrometry data extend observations from several other studies. Radiolabeling studies by Guarino and colleagues in 1976 investigated the tissue distribution of MeHg in American lobsters six days after ingesting food containing radioactive C14 MeHg [1]. They found that most of the radioactive Hg (68%) was retained in the hepatopancreas (tomalley) and only 8% in tail tissue. However, if a lobster was exposed to ambient water containing radiolabeled MeHg, 23% of the Hg was retained in tomalley, while 50% was retained in tail muscle. Hence, the uptake of MeHg in tomalley and tail depends on the source of Hg exposure. Because our study shows that Hg concentration in ambient water from the Gulf of Maine is negligible (see above), most of the Hg detected in tomalley and tail is expected to originate from ingestion.

A more recent study examining the biological fate of environmental Hg found that the total Hg level in Norwegian lobster (*Nephrops norvegicus*, harvested off the coast of Eastern Italy) was higher in the tail muscle of cooked lobsters (0.75 ppm) compared to raw tail muscle (0.51 ppm) [22]. The authors concluded that boiling likely increases the sensitivity of Hg detection due to the denaturation of astaxanthin, which releases its bound Hg [24]. This finding suggests that previous studies may have underestimated Hg levels, as toxicity assessments are typically performed on raw muscle tissue. Future paired comparisons of raw and boiled tissues from the same individuals would be informative.

### 3.4. Analysis of Hg levels in lobsters during early and late seasons

**Figure 8 •:**
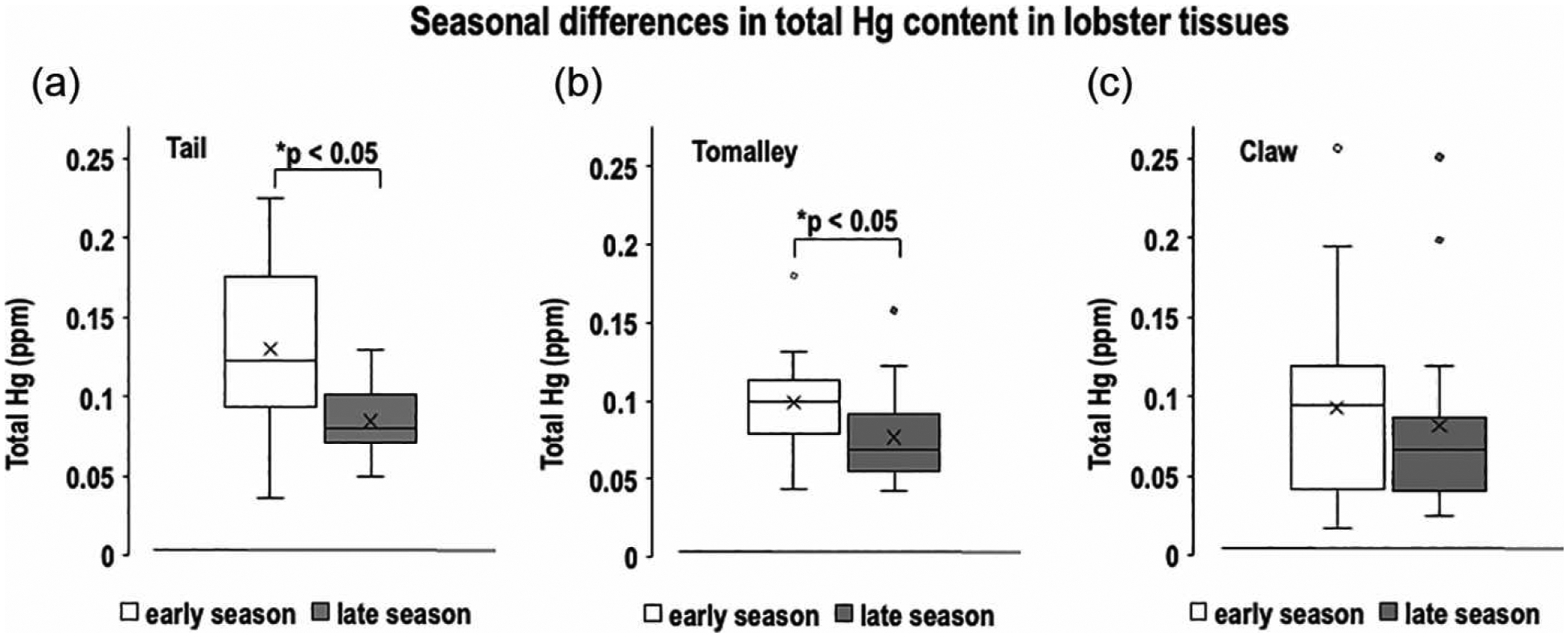
Comparison between mean total Hg in early and late seasons determined for tail (a), tomalley (b), and claw (c) of American lobsters collected in Casco Bay, ME. Middle horizontal lines in the gray boxes represent medians, “x” represents means, whiskers represent ± standard error, and the small circles outside of the boxes represent outliers.

To address our main hypothesis—that there are seasonal differences in Hg content—we analyzed the mean total Hg levels in tail, tomalley, and claw tissues during the early and late fishing seasons (Figure 8). The results show that early-season lobsters had significantly higher total Hg levels in their tails, with a 54% increase (0.130 ppm) compared with late-season lobsters (0.0844 ppm; p < 0.05) (Figure 8a). The average total level of Hg in tomalley of lobsters harvested in the early season was also significantly higher at 0.099 ppm versus 0.0766 ppm in the late season (29% difference; p < 0.05) (Figure 8b). In contrast, there was a nonsignificant difference (~14%) in the level of total Hg when comparing claws of lobsters from early season (0.0925 ppm) versus late season (0.0812 ppm) (p > 0.05) (Figure 8c). Because lobsters can self-amputate and regenerate claws, it is uncertain whether claws have experienced the same level of Hg exposure in both seasons. Additionally, Hg values in claw and tail tissues from the same lobster may not be proportional. The biological variation introduced by claw amputation could limit our ability to establish significance for the observed ~14% increase in early-season lobsters, particularly if catch-and-release methods contribute to widespread claw damage. More importantly, the significantly higher Hg levels in both tail and tomalley tissues from early-season lobsters confirm that total Hg content varies seasonally.

## 4. Discussion

One potential interpretation of our findings is that spring melt and runoff in the early season introduce an influx of Hg into the benthic habitat of nonmigratory lobsters. Altered Hg concentrations in the early season may also result from Hg sequestration by phytoplankton and zooplankton blooms in the circulating waters of Casco Bay. Subsequent consumption by higher trophic organisms, including lobsters, would lead to higher Hg concentrations in lobster tissues. The reduction of Hg in tail and tomalley tissue in the late-season lobster cohort could be due to the environmental depletion of Hg in the trophic system through organic dispersion or efflux into open waters. We were unable to validate this hypothesis because Hg levels in ambient water were below the level of detection, and we did not sample phytoplankton, zooplankton, or benthic substrates. We note that total precipitation in 2023 was 56.67 inches, making it the 13th wettest year on record since 1871. This increased precipitation could have contributed to higher Hg levels in the waters of Casco Bay, yet sampling future runoff sources would be necessary to confirm this mechanism. Multi-year studies will be required to assess whether the early to late seasonal variation in Hg levels observed in 2023 represents a sustained trend.

Seasonal differences in Hg levels may also be influenced by the migration of lobsters from offshore grounds with lower ambient Hg levels to inshore waters, combined with the selective trapping of nonmigratory lobsters early in the fishing season. The combination of trapping and lobster migration during the harvesting season would decrease the percentage of nonmigratory lobsters in the late-season cohort, leading to a lower mean total Hg content of tail tissue and tomalley. Evidence for migration could come from morphometric differences between the early- and late-season cohorts. However, there were no significant differences in any of the morphometric parameters tested (e.g., lobster weight, claw size, or shell size), suggesting that migration was not a dominant factor or resulted in the replacement of lobsters with a similar size range.

Several studies support the idea that lobster migration may influence Hg measurements throughout the season. Multiple tag-and-recapture studies have shown that lobsters migrate from offshore grounds to inshore waters in the summer and fall and return to deeper offshore waters in the winter and spring [26]. Cooper and colleagues estimated that 20–50% of the offshore lobster population is migratory. In addition, Skud and Perkins [27] demonstrated that lobsters migrate more and travel further as they develop longer legs. It is reasonable to assume that smaller lobsters may have higher Hg levels if they remain in a contaminated area for an extended period. Sampling tail muscles, Azad and colleagues reported that European lobsters trapped in the outer waters of Hardanger fjord (Norway) had 31% (0.19 ppm) of the amounts detected in lobsters from inner waters (0.62 ppm). The authors attributed this variation to several factors, including diet, ecosystem methylation potential, and species migration patterns [28].

The reasons for detecting seasonal differences in Hg content in lobsters from Maine’s inshore coastal waters remain unresolved in this study but are most likely multifactorial. While our results neither confirm nor rule out the effects of migration, we favor the primary conclusion that increased environmental exposure due to elevated concentrations of MeHg (in ambient water and diet) early in the season leads to higher total Hg levels in multiple tissues. Decreased Hg content later in the season may result from the environmental dispersal of Hg in open waters, the selective harvest of nonmigratory lobsters in the early season, and/or the migration of offshore lobsters to inshore habitats. Obtaining robust age-to-size ratios for this species would help characterize the potential effect of seasonality on MeHg accumulation. However, estimating the age and growth of American lobsters is challenging because (a) molting limits the availability of hard tissues for long-term measurement and (b) environmental conditions (e.g., temperature) influence growth rates. Nevertheless, gastric mill bands were recently used to estimate the ages of legal-size lobsters in the Gulf of Maine at 7.5 (±1.6) years old [29]. Given that American lobsters can live for more than 50 years, it is notable that the levels of MeHg detected in our study were observable in animals with less than a decade of life. Sampling larger individuals would yield interesting perspectives on the potential for long-term MeHg accumulation in older lobsters over subsequent decades.

Studying Hg levels in American lobsters is important not only for environmental research and lobster biology but also for the safety of seafood consumers worldwide. Only 8.8% (3/34) of the lobsters studied had total Hg levels exceeding the safety threshold of 0.2 ppm, all of which were trapped early in the season. Our study demonstrates that Maine lobsters are safe for consumption, although a small percentage of lobsters harvested early in the season may contain total Hg levels above the state-recommended safety level. Notably, the European Union has a less stringent Hg threshold of 0.5 ppm for the consumption of Norwegian lobster, and all lobsters we collected would meet this EU safety standard.

## 5. Conclusions

Pollution, climate change, and industrialization have created new challenges for coastal fisheries, making continuous monitoring essential to support the U.S. economy and ensure seafood safety worldwide. Our case study of lobsters from the coastal waters of Maine provides insights into the seasonal variation of Hg content in lobster tissues. This study presents a comprehensive chronological analysis of total Hg levels in lobsters throughout a fishing season. Our results indicate that Hg levels in lobsters vary depending on when they are trapped—early versus late fishing season. Therefore, Hg levels in lobster tissues are not static but rather a dynamic parameter that must be analyzed throughout the year. These findings suggest that the current state policy of examining lobster Hg content only twice per decade should be reconsidered.

Our study also emphasizes the importance of sample handling and tissue harvesting procedures in obtaining reliable Hg measurements. We recommend protein denaturation through boiling to increase the sensitivity of Hg measurements in lobster tail muscle, although boiling may have different effects on tomalley. Bioaccumulation of Hg occurs in all examined lobster tissues. While tomalley supports digestion and serves as the initial reservoir for ingested Hg [1], it is not the primary storage organ. Instead, our findings suggest that boiled lobster tail provides a more reliable tissue source for Hg measurement.

A positive takeaway from this study is that the Hg concentrations measured have primarily academic value for understanding seasonal environmental factors affecting lobsters. From a food safety perspective, all lobster specimens (n = 34) examined in this study meet the rigorous EU standards for Hg content in seafood (Hg < 0.5 ppm), with only three specimens showing a slight elevation (~0.2 ppm) above the more stringent U.S. threshold (Hg < 0.2 ppm). Given the extensive industrial Hg pollution in coastal waters over the past century, our findings suggest that Hg contamination in the Gulf of Maine has sufficiently abated, fostering a healthy marine ecosystem yielding lobsters that consistently meet stringent seafood safety standards.

## Data Availability

Data supporting these findings are available within the article, at https://doi.org/10.20935/AcadBiol7544, or upon request.
